# Diagnosis and Management Approaches for Cerebellar Hydatid Cysts: A Systematic Review of Cases

**DOI:** 10.7759/cureus.59706

**Published:** 2024-05-05

**Authors:** Jaber H Jaradat, Ibraheem M Alkhawaldeh, Abdulqadir J Nashwan, Yousef Al-Bojoq, Monther N Ramadan, Ibrahem Albalkhi

**Affiliations:** 1 School of Medicine, Mutah University, Al Karak, JOR; 2 Department of Nursing, Hamad Medical Corporation, Doha, QAT; 3 College of Medicine, Alfaisal University, Riyadh, SAU

**Keywords:** management, diagnosis, echinococcus, cerebellum, risk factor, cerebellar hydatid cysts, systematic review

## Abstract

Cerebellar hydatid cysts are uncommon lesions, with limited cases reported in the literature. This systematic review aimed to summarize current diagnostic and management approaches, given the low suspicion index of hydatid cysts in the cerebellum. The review was registered in the International Prospective Register of Systematic Reviews (PROSPERO) under registration number CRD42023437853. This study followed the Preferred Reporting Items for Systematic Reviews and Meta-Analyses (PRISMA-P) reporting guidelines. Two independent researchers searched PubMed, Scopus, and Google Scholar databases on June 27, 2023. We included 15 studies published between 1965 and 2022, comprising 12 case reports and three case series. A pooled analysis of reported cases (nine females and seven males) with cerebellar hydatid cysts revealed a mean age of 24 ± 20 years. Most of the cases were reported in Turkish hospitals (*n* = 8). The prominent signs and symptoms observed were headaches (10, 62.5%), ataxic gait (9, 56.25%), and visual disturbances (9, 56.25%). The time from symptom onset to hospital visit varied, with most patients seeking medical attention within the first three months. The left cerebellar hemisphere was the most common location of the cysts (6, 37.5%), and compression of the fourth ventricle was frequently observed. Computed tomography (CT) and magnetic resonance imaging (MRI) were the primary diagnostic tools used in three-fourths of cases, and surgical intervention was the primary treatment approach. Albendazole and praziquantel were commonly prescribed postoperatively, and two patients underwent preoperative needle decompression. This systematic review contributes to a better understanding of cerebellar hydatid cysts and guides future research and clinical management of this entity.

## Introduction and background

The prevalence and incidence of hydatid cysts and echinococcosis among zoonoses is increasing worldwide. The incidence of the main genus Echinococcus granulosus ranges from 1 to 220 cases per 100,000 inhabitants in endemic areas [1]. A high prevalence has been reported in the Mediterranean, Russian Federation, China, Africa (Northern and Eastern Regions), Australia, and South America. The main sites for primary hydatidosis are the lungs and liver, but they can occur in any organ, including the brain, in 1% to 2% of all hydatidosis cases [1]. Children are affected by 50% to 75% of intracranial hydatid cysts. Most recorded cases of intracranial hydatid cysts are located in cortical areas, specifically the parietal lobe. The mainstay of management involves surgical removal using the water-jet dissection technique [2]. However, posterior fossa lesions are rare, and there is a paucity of literature on this topic. These cases have a unique course and treatment because of their proximity to several neuronal structures, such as the brainstem [3]. The number of reported cases of cerebellar hydatid cysts with varying management approaches and high misdiagnosis rates remains unknown. This study aimed to provide a comprehensive overview of the patient characteristics, including symptoms, signs, complications, and strategies to manage these cases.

## Review

Methodology

This review was registered at the International Prospective Register of Systematic Reviews (PROSPERO) under CRD42023437853. This review followed the Preferred Reporting Items for Systematic Reviews and Meta-Analyses (PRISMA-P) guidelines [4].

Search Strategy

Two independent researchers performed a search on June 27, 2023, using PubMed, Scopus, and Google Scholar. The search string used for each database included the Medical Subject Heading (MeSH) database or other subject terms, search filters, and synonyms to optimize the keyword selection. The search strategy was peer-reviewed according to the PRESS (Peer Review of Electronic Search Strategies) guidelines [5].

The search terms used were as follows: ("Echinococcoses" OR "Echinococcus Infection" OR "Echinococcus Infections" OR "Infection, Echinococcus" OR "Cystic Echinococcosis" OR "Cystic Echinococcoses" OR "Echinococcoses, Cystic" OR "Echinococcosis, Cystic" OR "Hydatidosis" OR "Hydatidoses" OR " Cystic Echinococcosis " OR "Cyst, Hydatid Diseases" OR "Echinococcus Granulosus Infection" OR "Echinococcus Granulosus Infections" OR "Granulosus Infection, Echinococcus" OR "Granulosus Infections, Echinococcus" OR "Infection, Echinococcus Granulosus" OR "Infections, Echinococcus Granulosus") AND ("Cerebellums" OR "Corpus Cerebelli" OR "Cerebella" OR "Parencephalon" OR "Parencephalons"). The full search terms used for each database are presented in Appendix A.

Furthermore, the reference lists of the included studies were manually checked, and backward citation analysis was conducted. In case of missing data or if the full text is unavailable, the author/s were contacted via email to seek further clarification. Additionally, PROSPERO was checked using the terms (“Cerebellum”) AND (“Hydatid cyst”) to identify any ongoing studies. No time restrictions were applied and only English articles were searched.

Study Selection Criteria

We identified original case reports and case series as eligible study designs for our systematic review while excluding editorials, review articles, and other publications that did not report any primary data. Participants of any age, sex, ethnicity, or comorbidity with hydatid cysts in the cerebellum or cerebellar region were considered relevant for this review. Eligible studies were limited to those published in English.

Screening and Data Extraction

Studies retrieved from all databases were exported to Rayyan.ai, an online tool to identify and eliminate duplicate records [6]. Subsequently, two independent authors assessed the remaining publications' titles, abstracts, and keywords to determine their eligibility for inclusion in this review. Then, the full texts of the publications that met the initial screening criteria underwent a thorough review to confirm their eligibility for inclusion in the review. Discrepancies were resolved by consensus or consultation with a third reviewer. Data extraction was performed independently by the same two reviewers using a standardized spreadsheet. The extracted data included study characteristics (author, year, country, and study design), participant characteristics (sample size, age, sex, and comorbidities), intervention/exposure, outcomes, and relevant findings related to the factors and risks associated with developing cerebellar hydatid cysts. Considering the heterogeneity of the included studies, we performed a narrative synthesis of the findings. The extracted data are presented in a tabular format, summarizing the factors and risks associated with the development of cerebellar hydatid cysts.

Quality Assessment and Risk of Bias

We assessed the risk of bias in the included studies using The Joanna Briggs Institute Critical Appraisal tools (JBI) (Appendix B). JBI assesses the methodological quality of the included studies. Disagreements were resolved by consensus or consultation with a third reviewer. However, we did not consider the quality of the study to be an inclusion criterion, and we included all studies with diverse quality scores.

Statistical Analysis

Data management and cleaning were conducted for all patients across the 15 case reports and series. Pooled descriptive analyses using frequencies and proportions were performed using IBM SPSS Statistics for Windows, Version 28.0 (IBM Corp., Armonk, NY).

Results

Characteristics of Included Studies

**Figure 1:**
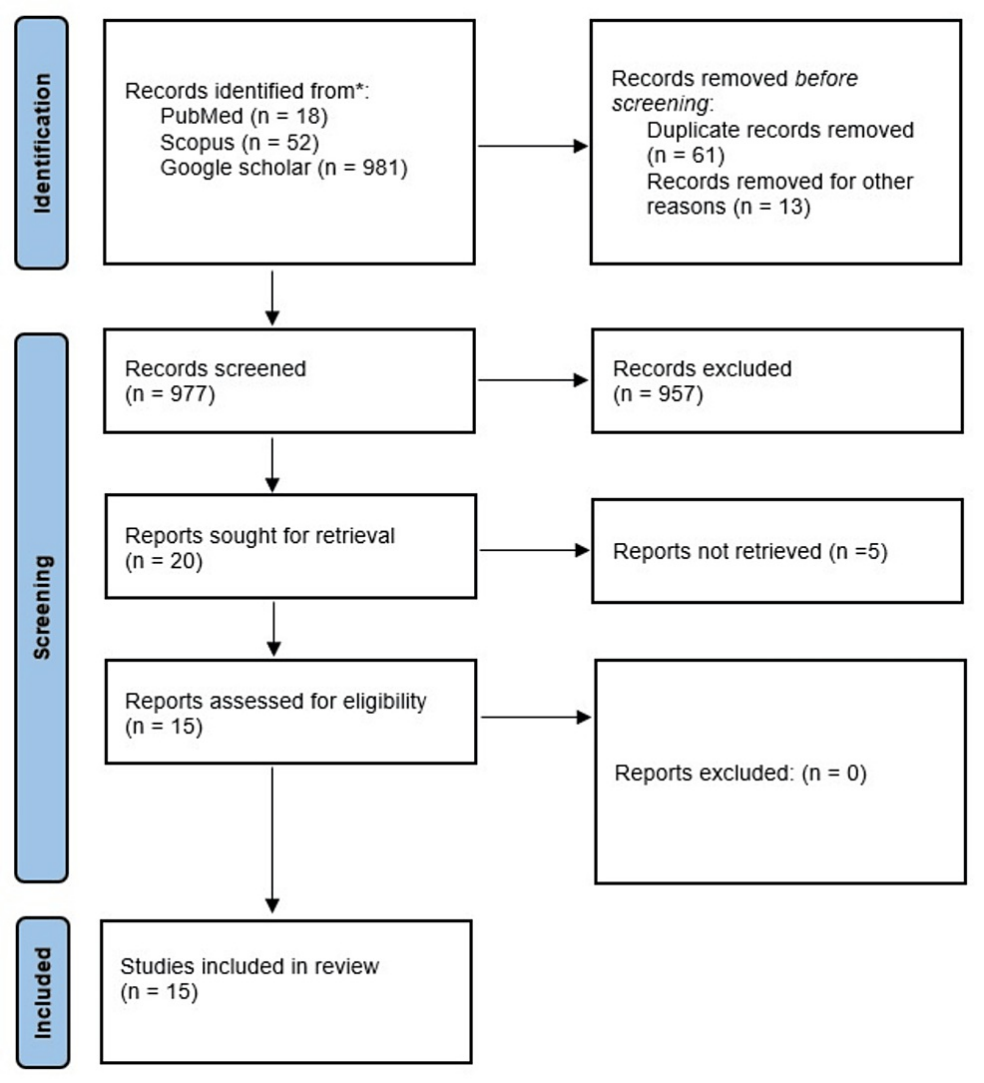
Summary of study selection following the PRISMA flowchart. PRISMA, Preferred Reporting Items for Systematic Reviews and Meta-Analyses

The literature search yielded 1,051 articles, of which 61 were duplicates and 13 were removed for other reasons. Accordingly, 977 articles were screened in their title/abstract form and 957 were excluded for being irrelevant (not meeting the inclusion criteria). The remaining 20 articles were tested against the inclusion criteria in their full-text form, and five articles were excluded because of the unavailability of the full text. Finally, 15 articles were included in this systematic review, 12 of which were case reports and 3 case series were published between 1965 and 2022. Figure 1 shows a flowchart of the selection process. Table 1 presents the characteristics of the included studies.

**Table 1:** Summary of the included cases.

Study ID	Year	Design	Number of Participants	Gender	Age (years)	Country
Samiy and Zadeh [7]	1965	Case series	9 (1)	Female	27	Iran
Copley et al. [8]	1992	Case series	3 (1)	Female	4	Republic of South Ahca
Beşkonakli et al. [9]	1996	Case report	1	Female	15	Turkey
Yasha et al. [10]	2006	Case report	1	Male	13	India
Akdemir et al. [11]	2007	Case report	1	Male	19	Turkey
Kayaoglu [12]	2008	Case report	1	Male	10	Turkey
Is et al. [13]	2009	Case report	1	Female	13	Turkey
Ozdol et al. [14]	2011	Case report	1	Male	23	Turkey
Fakhouri et al. [15]	2015	Case report	1	Female	5	Syria
Taghipour et al. [3]	2017	Case report	1	Female	62	Iran
Karthigeyan et al. [16]	2019	Case report	1	Male	Young	India
Belfquih et al. [17]	2021	Case report	1	Male	49	Morocco
Elvan-Tuz et al. [18]	2021	Case series	3 (2)	Female	Case 1: 16; Case 2: 9	Turkey
Aryal et al. [19]	2022	Case report	1	Male	28	Nepal
Dere et al. [20]	2022	Case report	1	Female	68	Turkey

Pooled Analysis of Reported Cases

The study included a total of 16 patients (nine females and seven males) who had cerebellar hydatid cysts, with a mean age of 24.1 ± 20.0 years, with median (interquartile range or IQR) = 16 (11.5 -27.5). All were reported by hospitals, except for five cases, and most reported cases were from Turkey (*n* = 8). The most frequent signs and symptoms were headaches (10, 62.5%), ataxic gait (9, 56.25%), and visual disturbances (9, 56.25%). In addition, patients frequently experienced vomiting (6, 37.5%), cranial nerve palsies (6, 37.5%), and nausea (4, 25%). Other positive cerebellar signs, such as lethargy and depressed reflexes, were also reported (8, 50%) (Table 1). The period from the onset of symptoms to hospital intervention varied among participants, as most patients showed symptoms within the first three months (10, 62.5%), while others came within one year, except for one patient who had symptoms for 14 years before seeking intervention.

Most cysts were located in the left cerebellar hemisphere (6, 37.5%). In addition, compression was most frequently reported in the fourth ventricle, with only three cases of calcification. Medical history and comorbidities were reported in a limited number of cases. Previous liver and lung hydatid cysts were reported in three cases, suggesting the risk of cerebellar hydatid cysts in previously exposed individuals.

Cystic lymphangioma, cerebellar abscess, cerebellitis, metastatic cerebellar tumor, cystic cerebellar tumor, cerebellar tuberculoma, and encephalitis were the differential diagnoses in the reported cases. The first interactions for diagnosis were CT and MRI, both of which were used in 12 cases (75%), which were, in most cases, confirmed postoperatively with lab results. The investigations and laboratory findings were mostly consistent across the studies. Based on the reported data, surgical intervention was the main treatment model in all cases, with variable approaches according to the cyst site, frequent use of albendazole and praziquantel postoperatively, and only two cases reported preoperative needle decompression. The treatment combinations used are presented in Table 2. Follow-up was uneventful in most cases (14, 87.5%), except in two patients. The characteristics of the included studies are summarized in Table 1.

**Table 2:** Presents characteristics of the hydatid cysts. NR, not reported; HC, hydatid cyst; AZ, Albendazole; PZ, praziquantel; CP, cerebellopontine angle; IAC, internal acoustic canal; JF, Jugular foramen; FLAIR, Fluid-Attenuated Inversion Recovery; CSF, cerebrospinal fluid

Study ID	Location of the cyst	Compression location	Calcification location	Medical history and comorbidities	Misdiagnosis	MRI	CT	Treatment modality (postoperation)	Follow-up
Samiy and Zadeh (1965) [7]	Left cerebellum	NR	Posterior fossa	Impairment of vision and severe ataxia at the age of 14	Encephalitis at the age of 14 and left cerebellar tuberculoma at the age of 28	Not used	Not used	Surgical excision	Uneventful and improvements
Copley et al. (1992) [8]	Left cerebellum	Fourth ventricle	NR	NR	NR	Not used	Large, low-density, non-enhancing cyst	Surgical excision with post-op AZ, PZ, and dexamethasone	Uneventful and improvements
Beşkonakli et al. (1996) [9]	Right occipital extradural space extending to right infratentorial extradural region	Right lateral and 4^th^ ventricles with adjacent right hemispheric bone	NR	Normal	NR	Not used	Large cystic lesion	Surgical excision with post-op AZ	Uneventful and improvements
Yasha et al. (2006) [10]	Left cerebellum	Fourth ventricle	NR	NR	Cystic cerebellar tumor	Not used	Oval, hypodense, non-enhancing	Surgical excision	NR
Akdemir et al. (2007) [11]	Lateral CP cistern involving the right IAC and JF	NR	NR	Hepatic HC was excised two years ago	NR	Cranial cystic lesion	Normal cranial CT when diagnosed with a hepatic cyst	Surgical excision	Uneventful and improvements
Kayaoglu (2008) [12]	Posterior fossa	Fourth ventricle	No calcification	NR	NR	Spheroid cystic lesion: hypointense on T1 and hyperintense on T2, non-enhancing with contrast	Large cystic lesion	Surgical excision with post-op AZ and antibiotic	Uneventful and improvements
Is et al. (2009) [13]	Left cerebellum	Fourth ventricle	No calcification	NR	Cystic cerebellar tumor	Spherical cystic lesion: hypointense on T1 and hyperintense on T2, with contrast enhancement of the rim of the lesion	Cystic lesion	Surgical excision with post-op AZ	Uneventful and improvements
Ozdol et al. (2011) [14]	Left cerebellum	NR	Right liver and upper lobe of the lung	NR	Metastatic cerebellar tumor	Marked peripheral contrast enhancement	Normal CT of the abdomen and chest looking for the primary lesion	Surgical excision with post-op AZ and cefotaxime	Uneventful and improvements
Fakhouri et al. (2015) [15]	Right cerebellum, and two cysts in the right hepatic lobe	NR	NR	NR	NR	A cystic lesion in the posterior fossa, hypointense on T1, hyperintense on T2 with minimally enhancing cyst wall, and pericystic edema	A large, right-sided cerebellar cystic mass with mild surrounding edema, ventriculomegaly, and periventricular edema	Surgical excision with post-op AZ and steroids	Uneventful and improvements
Taghipour et al. (2017) [3]	Left Meckel’s cave extending to the left CP angle and the middle fossa lateral to the left cavernous sinus	NR	NR	Diabetic neuropathy involving the cranial nerves	NR	Axial T2: a hyperintense round lesion on in left CP and middle fossa lateral to the cavernous sinus. Coronal T2: mass effect on the brain stem. Sagittal T1: enhanced gadolinium demonstrating both components without enhancement	Not used	Preoperative cyst aspiration and microsurgical excision with AZ post-op	Uneventful and improvements
Karthigeyan et al. (2019) [16]	Intra-axial CL surfacing over the right cerebellum	NR	NR	NR	NR	Right cerebellar cyst: spherical, smooth-walled, non-enhancing, and demonstrating signal intensity like CSF	A hypodense lesion like CSF intensity	Surgical excision with post-op PZ and AZ	Uneventful and improvements
Belfquih et al. (2021) [17]	Left CP cistern	Brain stem and fourth ventricle	NR	NR	Arachnoid cysts and epidermoid cysts	Hypointense cystic lesion on T1 and hyperintense on T2, enhancing wall with pericystic edema	Not used	Microsurgical excision with post-op AZ	Cranial nerve abnormalities improved markedly in this period
Elvan‑Tuz et al. (2021) [18]	Case 1: right mastoid cavity and cerebellar hemisphere. Case 2: left cerebellum	Case 1: NR Case 2: NR	Case 1: NR Case 2: left cerebellum	Case 1: Lung and liver HC three years ago and had operated three times	Case 1: NR Case 2: cerebellar abscess and cerebellitis	Case 1: T2-weighted, multiloculated cystic lesions on the right mastoid cavity and cerebellar hemisphere. Case 2: homogeneous, non-enhancing expansile cystic lesions in the left cerebellar hemisphere.	Case 1: Erosive and lytic bone changes Case 2: Not used	Case 1: Posterior fossa surgery and duraplasty with AZ post-op. Case 2: Posterior fossa surgery and duraplasty with AZ post-op. Antiedema treatment for cerebral edema.	Case 1: Uneventful & improved Case 2: Patient presented with vomiting. Swollen veins and increased tortuosity in both optic disk margins were observed. Brain edema improved during follow-up
Aryal et al. (2022) [19]	Posterior fossa	Adjacent cerebellum	NR	NR	NR	Axial FLAIR: Complete suppression of cystic lesion in the posterior fossa, but no suppression in a large lobulated component. Postcontrast axial images: No enhancement. DWI: No diffusion restriction within lesions.	Normal in the chest and abdomen	Surgical excision and oral AZ	NR
Dere et al. (2022) [20]	Left CP angle	NR	NR	NR	NR	Hyperintense on T2 and hypointense on T1 images, a cystic mass identified in left CP, without perilesional edema.	Cystic mass in the left CP angel	Suboccipital craniotomy	Uneventful and improvements

Discussion

We report the first systematic review to provide a comprehensive overview of the current diagnostic and management strategies for cerebellar hydatid cysts. Most cases of central nervous system (CNS) echinococcosis, which represent 2%-3% of all hydatid cysts worldwide, affect children. In our review, we found seven occurrences of this condition in children. According to a previous systematic review of CNS hydatid cysts. Males have a higher prevalence of hydatid cysts than females; this may be because men work more in agriculture, which helps the condition spread. Additionally, because of hunting activities, males appear to come into contact with canines, such as dogs, wolves, and foxes, which are the specific hosts for Echinococcus. In contrast, no sex predominance was observed among the cerebellar cysts. More than one-third of CNS hydatid cyst cases have been reported in Turkey, which is consistent with our review of eight cases from Turkey [21].

Diagnosis of Cerebellar Hydatid Cysts

Diagnosing hydatid cysts can be challenging due to the nonspecific nature of imaging findings and the high rate of misdiagnosis, particularly when differentiating them from arachnoid cysts and epidermoid tumors, which also commonly occur in the posterior fossa [19]. Hydatid cysts typically exhibit a spherical, round shape and have content with an attenuation value consistent with cerebrospinal fluid (CSF) [19]. Notably, there was no contrast enhancement of the cyst wall, and perilesional edema was absent. Over time, these cysts have been observed to grow, leading to the compression and displacement of the surrounding brain parenchyma and neighboring structures. Consequently, patients may present with focal signs and/or symptoms indicative of raised intracranial pressure [22]. This highlights the importance of carefully evaluating imaging results and considering the clinical context to achieve an accurate and timely diagnosis. This ensures appropriate management and treatment for patients with cerebellar hydatid cysts.

There were six cases of misdiagnosis in the included cases. The main differential diagnosis was cerebellar tumors, followed by arachnoid cysts and inflammatory changes. Cerebellar tumors are rare in adults [23]; therefore, cerebellar hydatid cysts must be considered when diagnosing adults with a mass lesion in the cerebellum. However, the cerebellum is the most common site for tumors in children younger than 15 [23], so it might be difficult to suspect hydatid cyst lesions. Five patients were under 15 years of age, two were misdiagnosed with cerebellar tumors, and one had a cerebellar abscess with cerebellitis.

Management of Cerebellar Hydatid Cysts

Managing cerebellar hydatid cysts primarily involves complete surgical excision, which is curative for the condition [24-26]. Furthermore, the success of the surgical procedure depends on preventing incomplete excision and rupture during surgery, as these are the most common causes of recurrence and poor outcomes. One widely used surgical technique for resection is Dowling's method [27], which is known for its effectiveness. This procedure involves a frontoparietotemporal inverted U-incision that provides wide exposure. The dura was opened, and cortical dissection was performed. Using hydrodissection with saline, the cyst was carefully separated from the adjacent brain parenchyma until it was entirely removed with an intact capsule. To maintain appropriate positioning, the patient is placed in the left lateral position, and the head of the operating table is slightly tilted downward. After cyst removal, the cavity was filled with isotonic saline, and duraplasty was performed to ensure proper closure and healing [27]. Lastly, for cases in the cerebellopontine angle and Meckles’ cave, it is recommended to perform preoperative needle aspiration along with microsurgical dissection of the cyst due to adhesion of the cyst to several neuronal structures, such as the brain stem and lower cranial nerve, which was performed in two cases in our review [3,17]. All cases in our systematic review utilized surgical excision, with no reported surgical complications.

Limitations

The limitations of this systematic review include the potential for publication bias, language bias, and variation in the quality of the included studies. Additionally, the review may be limited by the availability and quality of published literature on cerebellar hydatid cysts. We could not retrieve five studies, which adds to the limitations of this study. Furthermore, we excluded retrospective studies due to insufficient data. As most case reports are likely to focus on specific populations or healthcare settings, the generalizability of the findings to broader contexts may be limited. Future research should encourage multicenter collaboration and longitudinal studies to overcome these limitations and enhance the understanding of cerebellar hydatid cysts.

## Conclusions

This systematic review aimed to provide comprehensive insights into the factors and risks associated with cerebellar hydatid cyst development. Furthermore, we compared different treatments and diagnostic modalities. The findings of this review may contribute to a better understanding of the disease and guide future research, clinical practice, and public health strategies.

## Appendices

Appendix A: Search strategy

**Table 3:** Databases and keywords.

Database	Search Query
PubMed	("Echinococcoses" OR "Echinococcus Infection" OR "Echinococcus Infections" OR "Infection, Echinococcus" OR "Cystic Echinococcosis" OR "Cystic Echinococcoses" OR "Echinococcoses, Cystic" OR "Echinococcosis, Cystic" OR "Hydatidosis" OR "Hydatidoses" OR "Cysts, Hydatid" OR "Cyst, Hydatid" OR "Hydatid Cysts" OR "Hydatid Cyst" OR "Hydatid Disease" OR "Hydatid Diseases" OR "Echinococcus Granulosus Infection" OR "Echinococcus Granulosus Infections" OR "Granulosus Infection, Echinococcus" OR "Granulosus Infections, Echinococcus" OR "Infection, Echinococcus Granulosus" OR "Infections, Echinococcus Granulosus") AND ("Cerebellums" OR "Corpus Cerebelli" OR "Cerebella" OR "Parencephalon" OR "Parencephalons")
Google Scholar	("Echinococcoses" OR "Echinococcus Infection" OR "Echinococcus Infections" OR "Infection, Echinococcus" OR "Cystic Echinococcosis" OR "Cystic Echinococcoses" OR "Echinococcoses, Cystic" OR "Echinococcosis, Cystic" OR "Hydatidosis" OR "Hydatidoses" OR "Cysts, Hydatid" OR "Cyst, Hydatid" OR "Hydatid Cysts" OR "Hydatid Cyst" OR "Hydatid Disease" OR "Hydatid Diseases" OR "Echinococcus Granulosus Infection" OR "Echinococcus Granulosus Infections" OR "Granulosus Infection, Echinococcus" OR "Granulosus Infections, Echinococcus" OR "Infection, Echinococcus Granulosus" OR "Infections, Echinococcus Granulosus") AND ("Cerebellums" OR "Corpus Cerebelli" OR "Cerebella" OR "Parencephalon" OR "Parencephalons")
Scopus	TITLE-ABS-KEY(("Echinococcoses" OR "Echinococcus Infection" OR "Echinococcus Infections" OR "Infection, Echinococcus" OR "Cystic Echinococcosis" OR "Cystic Echinococcoses" OR "Echinococcoses, Cystic" OR "Echinococcosis, Cystic" OR "Hydatidosis" OR "Hydatidoses" OR "Cysts, Hydatid" OR "Cyst, Hydatid" OR "Hydatid Cysts" OR "Hydatid Cyst" OR "Hydatid Disease" OR "Hydatid Diseases" OR "Echinococcus Granulosus Infection" OR "Echinococcus Granulosus Infections" OR "Granulosus Infection, Echinococcus" OR "Granulosus Infections, Echinococcus" OR "Infection, Echinococcus Granulosus" OR "Infections, Echinococcus Granulosus") AND ("Cerebellums" OR "Corpus Cerebelli" OR "Cerebella" OR "Parencephalon" OR "Parencephalons"))

Appendix B: Quality assessment

**Table 4:** JBI quality assessment tool for case reports. JBI, Joanna Briggs Institute Critical Appraisal

Study ID.	Q1	Q2	Q3	Q4	Q5	Q6	Q7	Q8
(Samiy & Zadeh, 1965) [7]	Yes	Maybe	Yes	Yes	No	Yes	No	Yes
Copley et al., 1992) [8]	Yes	No	Yes	Yes	Yes	Yes	No	Maybe
(Beşkonakli et al., 1996) [9]	Yes	Maybe	Maybe	Yes	Yes	Yes	Yes	Yes
(Yasha et al., 2006) [10]	Maybe	No	Yes	Yes	Maybe	No	Yes	Yes
(Akdemir et al., 2007) [11]	Yes	Maybe	Yes	Yes	No	Yes	No	Yes
(Kayaoglu, 2008) [12]	Yes	No	Yes	Yes	Yes	Yes	No	Yes
(Is et al., 2009) [13]	Yes	No	Maybe	Yes	Yes	Maybe	No	Yes
(Ozdol et al., 2011) [14]	Yes	Yes	Yes	Yes	Yes	Yes	Yes	Yes
(Fakhouri et al., 2015) [15]	Yes	Maybe	Yes	Yes	Yes	Yes	No	Yes
(Taghipour et al., 2017) [3]	Yes	Yes	Maybe	Yes	Yes	Yes	Yes	Yes
(Karthigeyan et al., 2019) [16]	Maybe	No	Maybe	Yes	Yes	Yes	Yes	Yes
(Belfquih et al., 2021) [17]	Yes	No	Yes	Yes	Yes	Yes	Maybe	Yes
(Elvan‑Tuz et al., 2021) [18]	Yes	Yes	Yes	Yes	Yes	Yes	No	Yes
(Aryal et al., 2022) [19]	Yes	No	Yes	Yes	Yes	No	No	Yes
(Dere et al., 2022) [20]	Yes	No	Maybe	Yes	No	Yes	Maybe	Yes
